# The complete mitochondrial genome of *Alveopora japonica* (Scleractinia: Acroporidae)

**DOI:** 10.1080/23802359.2018.1501310

**Published:** 2018-08-28

**Authors:** In-Young Cho, Sung-Jin Hwang, Keun-Yong Kim, Moongeun Yoon, Il Hun Kim, Min-Seop Kim

**Affiliations:** a National Marine Biodiversity Institute of Korea, Seocheon, Republic of Korea;; b Department of Eco-Biological Science, Woosuk University, Jincheon-eup, Republic of Korea;; c Korea Maritime and Ocean University, Busan, Republic of Korea

**Keywords:** Mitochondrial genome, phylogeny, Alveopora japonica, Acroporidae

## Abstract

Here, for the first time, we sequenced the complete mitogenome of *Alveopora japonica* Eguchi, 1968 (Scleractinia: Acroporidae). Genome size was 17,886 bp with 13 protein-coding, two rRNA, and two tRNA genes. This gene composition was identical to the typical scleractinian pattern. Our results strongly support the recent transfer of this coral species to the family Acroporidae.

*Alveopora japonica* Eguchi 1968 is a zooxanthellate scleractinian coral belonging to the family Acroporidae. This species is generally distributed in high-latitude regions of East Asia, such as Korea (Jeju Island), Japan, and Taiwan (Denis et al. 2013; Matsumoto et al. 2016; Vieira et al. 2016). Growth forms of *A. japonica* are hemispherical or massive with ellipsoidal skeletons, and its colonies are dark green to brownish green (Veron and Stafford-Smith 2000). This species reproduces via hermaphroditic brooding, with oocytes and spermaries developing on separate mesenteries (Harii et al. 2001). *Alveopora japonica* is designated as Vulnerable on the IUCN Red List (IUCN 2008) and included in CITES Appendix II (CITES 1990) with other scleractinian corals. Their Threatened status is primarily because of the susceptibility to bleaching, harvesting for the aquarium trade, and typhoon-induced sedimentation or physical damage (Matsumoto et al. 2016). However, due to the effects of climate change, the population of *A. japonica* is actually increasing in Jeju Island in Korea, at odds with the general trend of coral populations (Denis et al. 2014).

In this study, an *A. japonica* specimen was collected from Jeju Island in 2017 and deposited in the National Marine Biodiversity Institute of Korea (MABIK CN00079315). Genomic DNA was extracted from the polyp tissue of this voucher specimen. Complete mitochondrial DNA was sequenced using long-range PCR and primer-walking method. Mitogenomic sequences of all species belonging to the Acroporidae were retrieved from GenBank and aligned with *A. japonica* mitogenome analysed in this study. Alignment of protein-coding genes accounted for amino-acid sequences and partitioned nucleotides into codons. Maximum-likelihood (ML) and Bayesian inference (BI) analyses were performed in RAxML 7.0.4 (Stamatakis 2006) and MrBayes 3.1.2 (Ronquist and Huelsenbeck 2003), respectively.

The complete *A. japonica* mitogenome is 17,886 bp in size (GenBank accession number MG851913), consisting of 14 protein-coding genes (*nad5-5'*, *nad1*, *cob*, *nad2*, *nad6*, *atp6*, *nad4*, *cox3*, *cox2*, *nad4l*, *nad3*, *nad5-5'*, *atp8*, *cox1*), two ribosomal RNA genes (*rrnL*, *rrnS*), and two transfer RNA genes (*trnM*, *trnW*). This composition is identical to the typical scleractinian mitogenome pattern.

Owing to the results from cladistics analyses (Fukuhori et al. 2005), the genus *Alveopora* was recently transferred from the Poritidae to the Acroporidae. Our phylogenetic tree (Figure 1) indicates that all genera of the Acroporidae, including *Alveopora*, are monophyletic. Thus, findings from this study strongly support the classification of *Alveopora* in the Acroporidae. We conclude that this novel report of the full *A. japonica* mitogenome sequence contributes valuable phylogenetic information on the Scleractinia.

**Figure 1.**
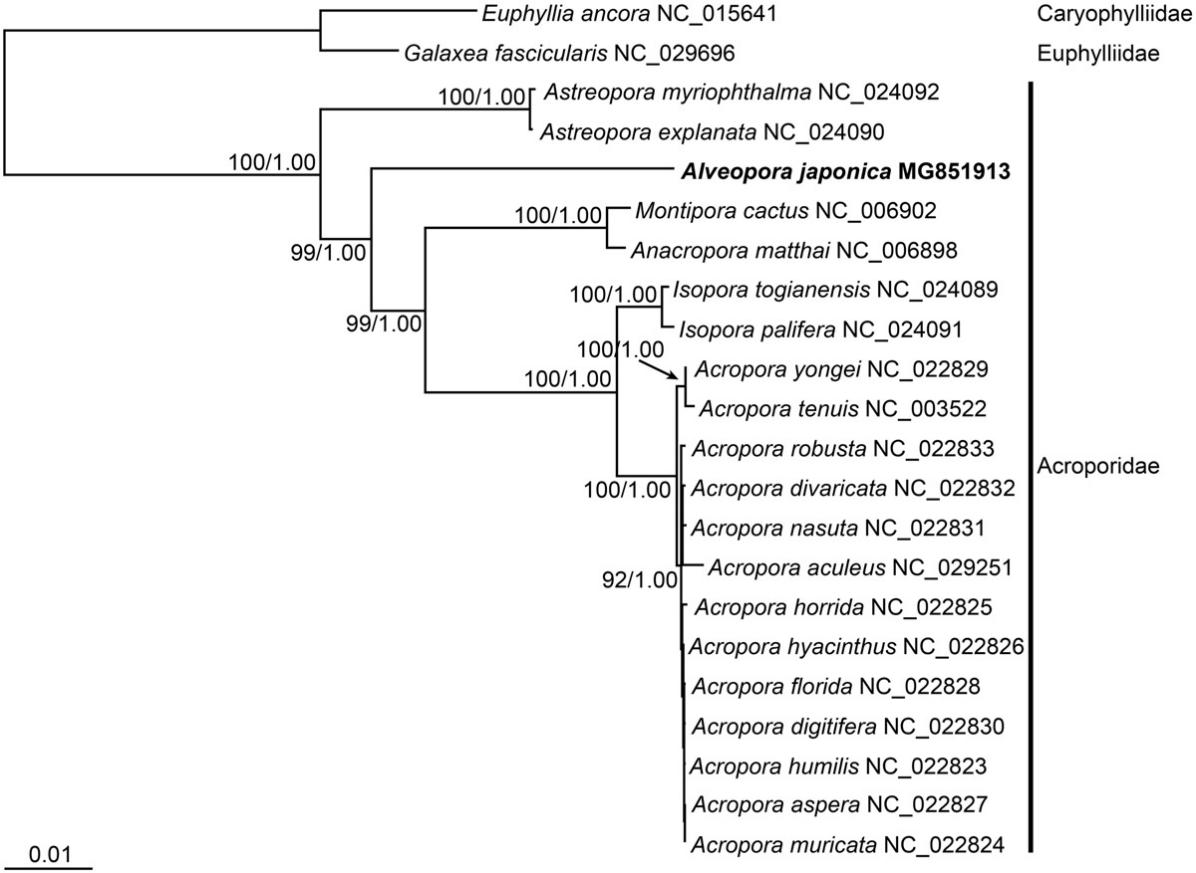
Maximum-likelihood (ML) tree inferred from the mitogenomic sequences of species in the Acroporidae. The sequence matrix used in the phylogenetic analyses consisted of codon positions from protein-coding genes. Bootstrap values >50% in the ML analysis and posterior probabilities >0.90 in the Bayesian inference analysis are indicated at each node. The mitogenome of Alveopora japonica, shown in bold, was sequenced for the first time in this study (GenBank accession number MG851913).
